# miR-23a-3p inhibits sepsis-induced kidney epithelial cell injury by suppressing Wnt/β-catenin signaling by targeting wnt5a

**DOI:** 10.1590/1414-431X2021e11571

**Published:** 2022-02-28

**Authors:** Junwei Ye, Huibing Feng, Zhiyong Peng

**Affiliations:** 1 Department of Critical Care Medicine, Zhongnan Hospital of Wuhan University, Wuhan, Hubei, China; 2 Department of Critical Care Medicine, Huangshi Central Hospital of Edong Healthcare Group, Hubei Polytechnic University, Huangshi, Hubei, China

**Keywords:** Acute kidney injury, miR-23a-3p, sepsis, Wnt/β-catenin pathway, wnt5a

## Abstract

The present study was designed to investigate the involvement of miR-23a-3p in the progression of sepsis-induced acute kidney injury (AKI). The expression levels of miR-23a-3p and wnt5a in sepsis-induced AKI patients and lipopolysaccharide (LPS)-treated HK-2 cells were detected by real-time PCR and western blotting. Then, the effects of miR-23a-3p overexpression on cell viability, apoptosis, and inflammatory cytokines secretion in LPS-stimulated HK-2 cells were investigated. Moreover, luciferase reporter assay was performed to confirm the regulatory relationship between miR-23a-3p and wnt5a. Whether miR-23a-3p regulated the activation of Wnt/β-catenin signaling was also explored. mR-23a-3p was lowly expressed in the serum of patients with sepsis-associated AKI and in LPS-treated HK-2 cells. In addition, the overexpression of miR-23a-3p restrained LPS-induced proliferation inhibition and promotion of apoptosis and cytokine production in HK-2 cells. Moreover, wnt5a was identified as a target of miR-23a-3p, which could be negatively regulated by miR-23a-3p. Overexpression of miR-23a-3p suppressed the activation of Wnt/β-catenin signaling in LPS-treated HK-2 cells, which was markedly reversed by wnt5a upregulation. Upregulation of miR-23a-3p may alleviate LPS-induced cell injury by targeting wnt5a and inactivating Wnt/β-catenin pathway, which may serve as a novel therapeutic target for sepsis-associated AKI.

## Introduction

Sepsis is a systemic inflammatory condition caused by the invasion of pathogens, leading to fatal multiple organ dysfunction syndrome (MODS) (1,2). The kidney is one of the most vulnerable target organs when sepsis occurs. Approximately 50% of patients with sepsis will develop acute kidney injury (AKI) that is characterized by the rapid decline and even loss of kidney function (3). Accordingly, AKI is a life-threatening complication of sepsis, which is implicated in increased morbidity and mortality in the intensive care unit (4,5). It is widely accepted that there are different pathophysiological mechanisms that lead to AKI (4). Many urinary and serum biomarkers have been described as increased in kidney injury, such as cystatin C, kidney injury molecule-1, among others, but they are not effective in reflecting kidney damage (6). In recent years, research has proposed that the release of inflammatory mediators, kidney hemodynamic abnormality, as well as microcirculation dysfunction and kidney cell apoptosis caused by kidney injury are involved in the pathogenesis of sepsis-associated AKI (7,8). Therefore, it is critical to identify potential biomarkers for early diagnosis, treatment, and prognosis of septic AKI.

Micro (mi) RNAs are a class of small non-coding RNAs composed of 19-25 nucleotides, which serve as post-transcriptional regulators of gene expression by binding to the target mRNA and inhibiting its translation (9). Since miRNA was first discovered in nematodes by Lee et al. (10), tens of thousands of miRNAs have been identified, and over 60% of human protein-coding genes are regulated by miRNAs (11). In mammals, miRNAs are widely involved in a variety of biological processes, such as cell differentiation, substance metabolism, tumor growth, and immune response, which are expected to become novel targets for the treatment of diverse diseases (12). In recent years, an extensive body of evidence suggests the involvements of several miRNAs in the progress of sepsis (13,14). For instance, Ge et al. (15) reported that miR-23a-3p expression was significantly downregulated in both the sepsis-induced AKI and sepsis-non AKI patients compared with the healthy controls. Nevertheless, to our knowledge, the physiopathological role of miR-23a-3p in sepsis-associated AKI remains elusive. It would be interesting to understand the relationship of miRNAs and mitochondrial oxidative stress and endothelial dysfunctions in sepsis and if they are related to early stages of this condition. The present study aimed to analyze the influence of miR-23a-3p in sepsis patients with AKI, and further investigate its regulatory pathway in lipopolysaccharide (LPS)-associated injury in kidney tubular epithelial cells.

## Material and Methods

### Patients and controls

Twenty-five sepsis patients with AKI (mean age=45.75 years, 15 females and 10 males) who fulfilled the definition and diagnostic criteria for sepsis proposed by the Society of Critical Care Medicine (SCCM) and the European Society of Intensive Care Medicine (ESICM) were enrolled in this study. The criteria for AKI were based on the Acute Kidney Injury Network (AKIN) classification (16,17). Patients were included if they: 1) were over 18 years old; 2) conformed to the diagnostic criteria of SCCM/ESICM; and 3) conformed to the diagnostic criteria of AKIN. The exclusion criteria were as follows: 1) patients aged less than 18 years or more than 80 years; 2) pregnant females; 3) patients with malignant tumors; 4) patients with chronic liver and kidney diseases, autoimmune diseases, and blood system diseases; and 5) patients treated with systemic hormones and immunosuppressive agents. In addition, 20 healthy volunteers (mean age=44.39 years, 12 females and 8 males) were recruited as healthy controls. The characteristics of the participants enrolled in the study are shown in Table 1. Written informed consents were obtained prior to blood sample collection. The experiments were performed with the approval of the Ethics Review Committee of Zhongnan Hospital of Wuhan University.

**Table 1:** Baseline characteristics of sepsis-induced acute kidney injury (AKI) patients (n=25) and healthy individuals (n=20).

Baseline characteristics	Sepsis-induced AKI	Healthy controls	P value
Age (years)	45.75±3.21	44.39±2.54	>0.05
Sex (males/females)	15/10	12/8	
Body mass (kg/m^2^)	26.23±7.54	25.99±9.28	>0.05
Blood urea nitrogen (mM)	10.25±1.56	5.23±1.77	<0.05
Serum creatinine (mM)	260.23±20.56	46.75±17.53	<0.05

Data are reported as means±SD, except for sex (n). Groups were compared with the Student's *t*-test.

### Cell culture, treatment, and transfection

Human kidney tubular epithelial cell line HK-2 purchased from Cell Bank of Chinese Academy of Sciences (China) was cultured in Dulbecco's modified Eagle's medium (DMEM) supplemented with 10% fetal bovine serum, L-glutamine, 100 U/mL penicillin, and 100 mg/mL streptomycin (all from Gibco, USA) in humidified air with 5% CO_2_ at 37°C. At 70% confluence, HK-2 cells were treated with 1 μg/mL lipopolysaccharide (LPS; Sigma, USA) for 24 h to induce the *in vitro* model of sepsis, as previously described (18,19). The miR-23a-3p mimics (miR-23a-3p), mimic scrambled control (miR-NC), anti-miR-23a-3p oligonucleotides, anti-miR negative control (anti-miR-NC), pcDNA3.1-wnt5a (wnt5a), and pcDNA3.1 empty vector (Vector) were bought from GenePharma (China). Cell transfection was performed using Lipofectamine 2000 reagents (Invitrogen, USA) before LPS stimulation according to manufacturer's instructions. Transfection efficiency was assessed by real-time PCR and western blotting.

### Luciferase reporter assay

The wild‐type or mutated wnt5a expression luciferase plasmids were co-transfected with miR-23a-3p mimics or miR-NC (negative control) into HK-2 cells using Lipofectamine 2000. After 48 h transfection, the dual-luciferase reporter system (Promega, USA) was used to detect the luciferase activity.

### Reverse transcription and quantitative polymerase chain reaction (RT-qPCR)

Trizol reagent (Invitrogen) was used to extract total RNA according to the manufacturer's instructions. PrimeScript RT reagent kit (Takara, China) was then applied for first-strand cDNA synthesis. The mRNA expression levels were detected by qPCR using the SYBR Green kit (Takara). The primers were miR-23a-3p (Forward: 5′‐CCAATTGCGCCTTCAGGCTA‐3′, Reverse: 5′‐CGGCAGAGTCCTTACCCACA‐3′); wnt5a (Forward: 5′-ATTCTTGGTGGTCGCTAGGTA-3′, Reverse: 5′-CGCCTTCTCCGATGTACTGC-3′), GAPDH (Forward: 5′‐CGCGAGAAGATGACCCAGAT‐3′, Reverse: 5′‐GGGCATACCCCTCGTAGATG‐3′); and U6 (Forward: 5′‐TCACTCTCAGAAGATCA‐3′, Reverse: 5′‐GGGACGGACACGGTTG‐3′). The results were normalized to GAPDH or U6, and the expression fold-change was calculated using the 2^-ΔΔCt^ method.

### Cell viability assay

Cell viability was determined using the CCK-8 kit (Dojindo Molecular Technologies, China). In brief, HK-2 cells were seeded onto 96-well plates at a density of 5×10^3^ cells per well and incubated with 10 µL CCK-8 solution at 0 and 24 h. The absorbance was then measured at 450 nm after 1 h incubation using a microplate reader (Bio-Rad Laboratories, USA).

### Cell apoptosis assay

Cell apoptosis was measured by flow cytometry with Annexin V-FITC/PI double staining. HK-2 cells were collected and washed with cold PBS buffer. Flow cytometry analysis was performed using Annexin V-FITC/PI apoptosis detection kit (eBioscience, USA). The positive cells were considered as apoptotic cells.

### Immunoblotting

The protein concentration was determined using a BCA protein assay kit (Thermo Scientific, China). Equal amounts of total protein were separated by SDS-PAGE, electroblotted on the polyvinylidene fluoride membranes (Millipore, USA), and immunoblotted with the primary antibodies (1:1000 dilution) against wnt5a, Bax, Bcl-2, cleaved caspase-3, β-catenin, c-myc, cyclin D1, and β-actin as a loading control, which were all obtained from Cell Signaling Technology (USA). The membranes were then incubated with horseradish peroxidase‐conjugated IgG antibodies for 1 h at room temperature. The protein levels were measured using an enhanced chemiluminescence kit (Millipore) through ImageJ software (NIH, USA).

### ELISA

The concentrations of interleukin (IL)-6, IL-1β, and tumor necrosis factor (TNF)-α in the culture supernatants were determined using commercial enzyme-linked immunosorbent assay (ELISA) kits (R&D Systems, USA) following the manufacturer's protocol. The absorbance was measured at 450 nm on a microplate reader. The caspase-3 activity was determined with the Caspase-3 Colorimetric Activity Assay kit (Keygen Biotech, China). Absorbance values at 405 nm were recorded.

### Statistical analysis

Data are reported as means±SD and analyzed by SPSS software v19.0 (IBM, USA). All data were checked for normality using Kolmogorov-Smirnov tests. Comparisons were performed using Student's *t*-test and one-way ANOVA. Correlation between expression levels of miR-23a-3p and wnt5a were performed using Spearman's rank correlation coefficient. Differences with P<0.05 (two-tailed) were considered statistically significant.

## Results

### miR-23a-3p was downregulated in sepsis patients and LPS-treated HK-2 cells

**Figure 1:**
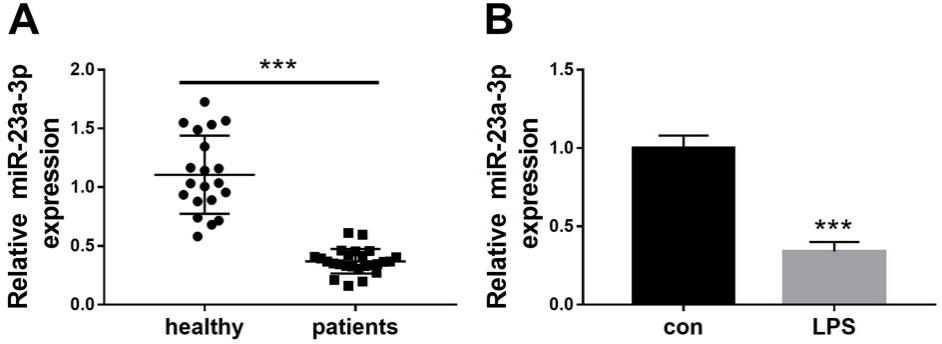
miR-23a-3p is downregulated in sepsis patients and lipopolysaccharide (LPS)-treated HK-2 cells. **A**, The expression of miR-23a-3p in serum of patients with sepsis-induced acute kidney injury (n=25) and healthy controls (n=20) was detected by RT-qPCR. **B**, The expression of miR-23a-3p in LPS-treated (1 μg/mL; 24 h) HK-2 cells and untreated cells (con) was detected by RT-qPCR. Data are reported as means±SD. ***P<0.001 (Student's *t*-test).

The expression levels of miR-23a-3p were initially measured in sepsis patients with AKI and healthy subjects using real-time PCR. The results demonstrated that miR-23a-3p expression was significantly decreased in serum of patients with sepsis-induced AKI compared to healthy controls (Figure 1A). miR-23a-3p expression was also suppressed in LPS-treated HK-2 cells compared to the untreated cells (Figure 1B).

### Overexpression of miR-23a-3p alleviated LPS-induced apoptosis and inflammation in HK-2 cells

**Figure 2:**
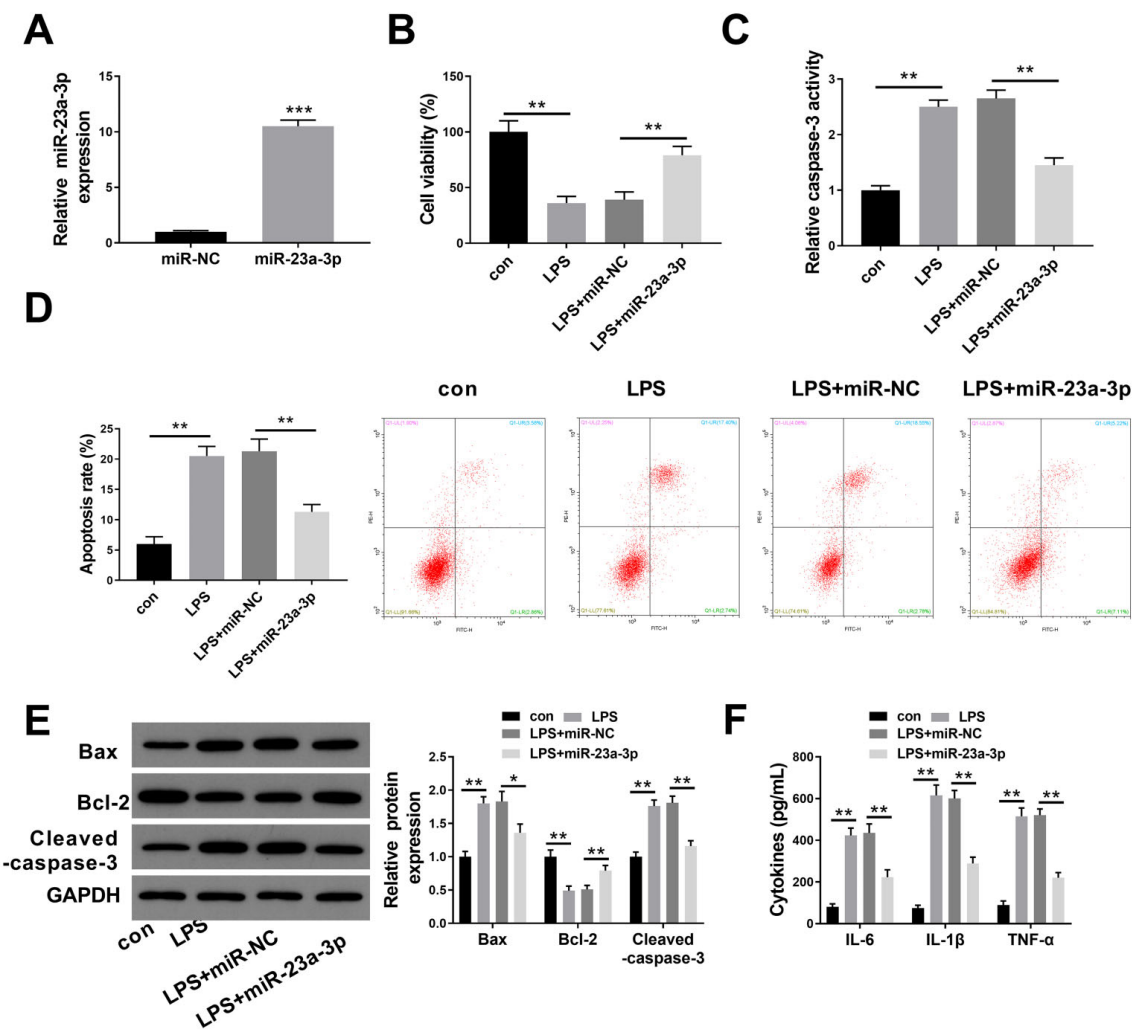
Overexpression of miR-23a-3p alleviates lipopolysaccharide (LPS)-induced apoptosis and inflammation in HK-2 cells. **A**, RT-qPCR was conducted to detect the expression levels of miR-23a-3p in HK-2 cells after the overexpression of miR-23a-3p. **B**, CCK-8 assay was used to detect the proliferation of HK-2 cells induced by LPS after the overexpression of miR-23a-3p. **C**, The colorimetric assay was carried out to assess the caspase-3 activity in HK-2 cells induced by LPS after the overexpression of miR-23a-3p. **D**, Flow cytometry was applied to evaluate the apoptotic rate of HK-2 cells induced by LPS after the overexpression of miR-23a-3p. **E**, Western blotting was done to detect the protein levels of Bax, Bcl-2, and cleaved caspase-3 in HK-2 cells induced by LPS after the overexpression of miR-23a-3p. **F**, ELISA was performed to estimate the production of interleukin (IL)-6, IL-1β, and tumor necrosis factor (TNF)-α in HK-2 cells induced by LPS after the overexpression of miR-23a-3p. The results are representative of three independent experiments (n=3 per group). Data are reported as means±SD. *P<0.05; **P<0.01; ***P<0.001 (ANOVA). con: control; NC: negative control.

To explore the function of miR-23a-3p in LPS-treated HK-2 cells, we established a miR-23a-3p overexpression model in HK-2 cells by transfection with miR-23a-3p mimics prior to LPS stimulation, and the upregulation efficacy was confirmed by real-time PCR (Figure 2A). The effects of miR-23a-3p overexpression on cell proliferation, apoptosis, and inflammatory response in LPS‐stimulated HK‐2 cells were further determined. The results of CCK-8 cell viability assay showed that compared to the untreated HK-2 cells, LPS treatment reduced the cell viability, while the overexpression of miR-23a-3p markedly increased the viability of LPS‐stimulated HK-2 cells (Figure 2B). The treatment of HK-2 cells with LPS produced higher apoptotic rate as evidenced by the results of flow cytometry, while upregulation of miR-23a-3p significantly attenuated cell apoptosis (Figure 2C). Western blot analysis indicated that LPS-treated HK-2 cells showed higher levels of proapoptotic proteins Bax and cleaved caspase-3 and lower levels of antiapoptotic protein Bcl-2 than those of untreated cells, while the enforced expression of miR-23a-3p exhibited reverse effects of the protein levels of these apoptosis-associated proteins (Figure 2D). Consistently, the colorimetric assay revealed that enhanced caspase-3 activity was observed in LPS-treated HK-2 cells, which was inhibited by miR-23a-3p overexpression (Figure 2E).

ELISA indicated that LPS treatment induced higher inflammatory cytokines, including IL-6, IL-1β, and TNF-α in HK-2 cells, which was significantly reversed by miR-23a-3p upregulation (Figure 2F).

### miR-23a-3p directly targeted and negatively regulated wnt5a in HK-2 cells

**Figure 3:**
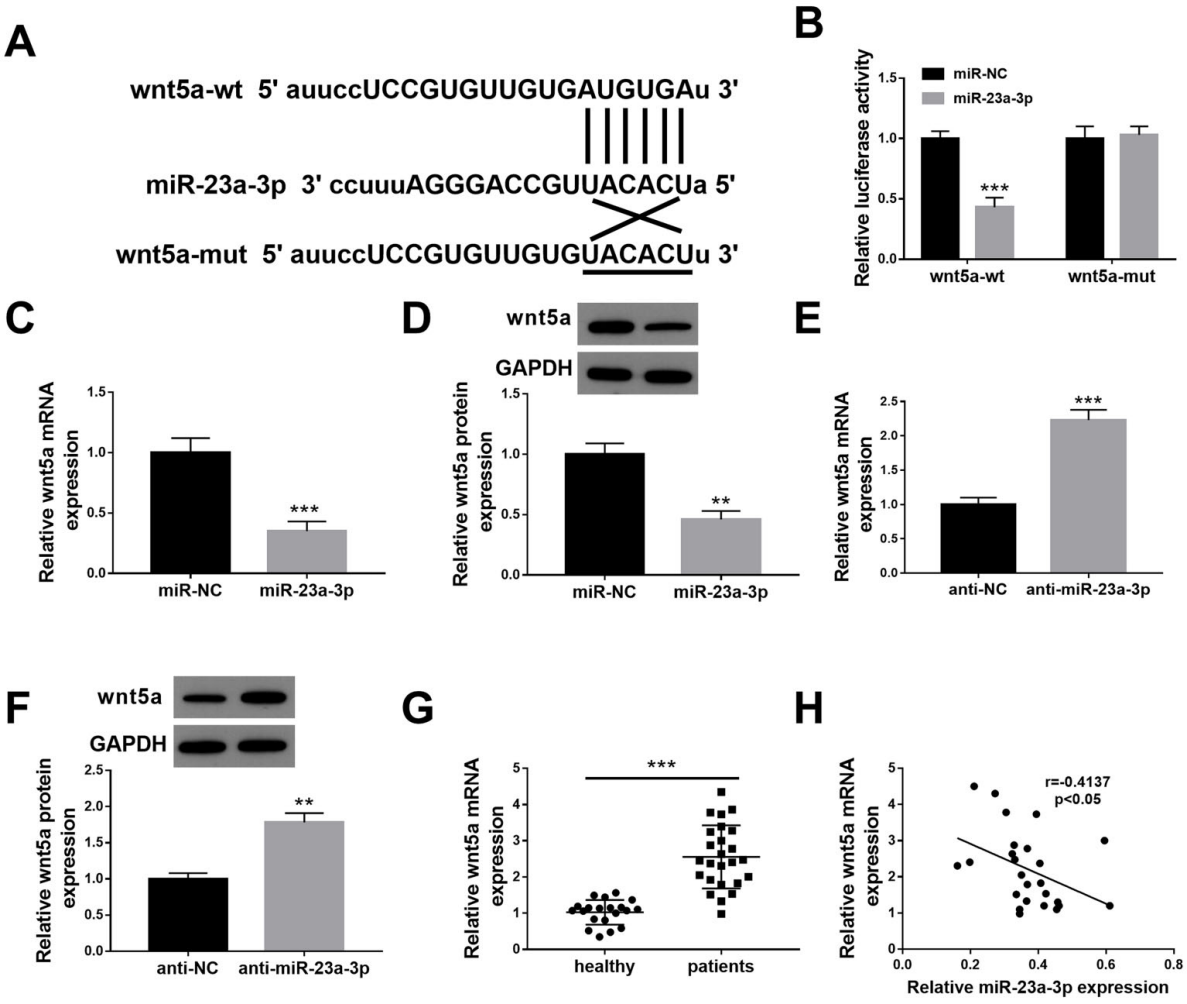
miR-23a-3p directly targets and negatively regulates wnt5a in HK-2 cells. **A**, The base complementary pairing relationship between miR-23a-3p and wnt5a. **B**, Dual luciferase assay was performed to determine the targeting relationship of miR-23a-3p and wnt5a. **C** and **D**, RT-qPCR and western blotting were performed to detect the mRNA and protein levels of wnt5a in HK-2 cells after the overexpression of miR-23a-3p. **E** and **F**, RT-qPCR and western blotting were performed to detect the expression of wnt5a in HK-2 cells after the knockdown of miR-23a-3p. **G**, The mRNA expression of wnt5a in serum of patients with sepsis-induced AKI (n=25) and healthy controls (n=20) was detected by RT-qPCR. **H**, The correlation between miR-23a-3p and wnt5a expression in serum of patients with sepsis-induced acute kidney injury was analyzed using Spearman's rank correlation coefficient. The results are representative of three independent experiments (n=3 per group). Data are reported as means±SD. **P<0.01; ***P<0.001 (Student's *t*-test). NC: negative control; wt: wild-type; mut: mutated.

We next elucidated the possible downstream targets of miR-23a-3p. Based on starBase software (https//bio.tools), the predicted binding sequences of miR-23a-3p and wnt5a is shown in Figure 3A. Luciferase assay in HK-2 cells showed that the luciferase activities of wnt5a were significantly reduced by miR-23a-3p mimics (Figure 3B). As expected, the mRNA and protein levels of wnt5a were significantly downregulated in miR-23a-3p-overexpressed HK-2 cells (Figure 3C and D). HK-2 cells were transfected with miR-23a-3p inhibitor to suppress the miR-23a-3p expression. Subsequent experiments revealed that miR-23a-3p knockdown led to the upregulation of wnt5a expression at mRNA and protein levels (Figure 3E and F). The mRNA expression of wnt5a was significantly upregulated (Figure 3G) and negatively correlated with miR-23a-3p expression (Figure 3H) in serum of sepsis-induced AKI patients.

### miR-23a-3p attenuated LPS-induced HK-2 cell injury by downregulating wnt5a

**Figure 4:**
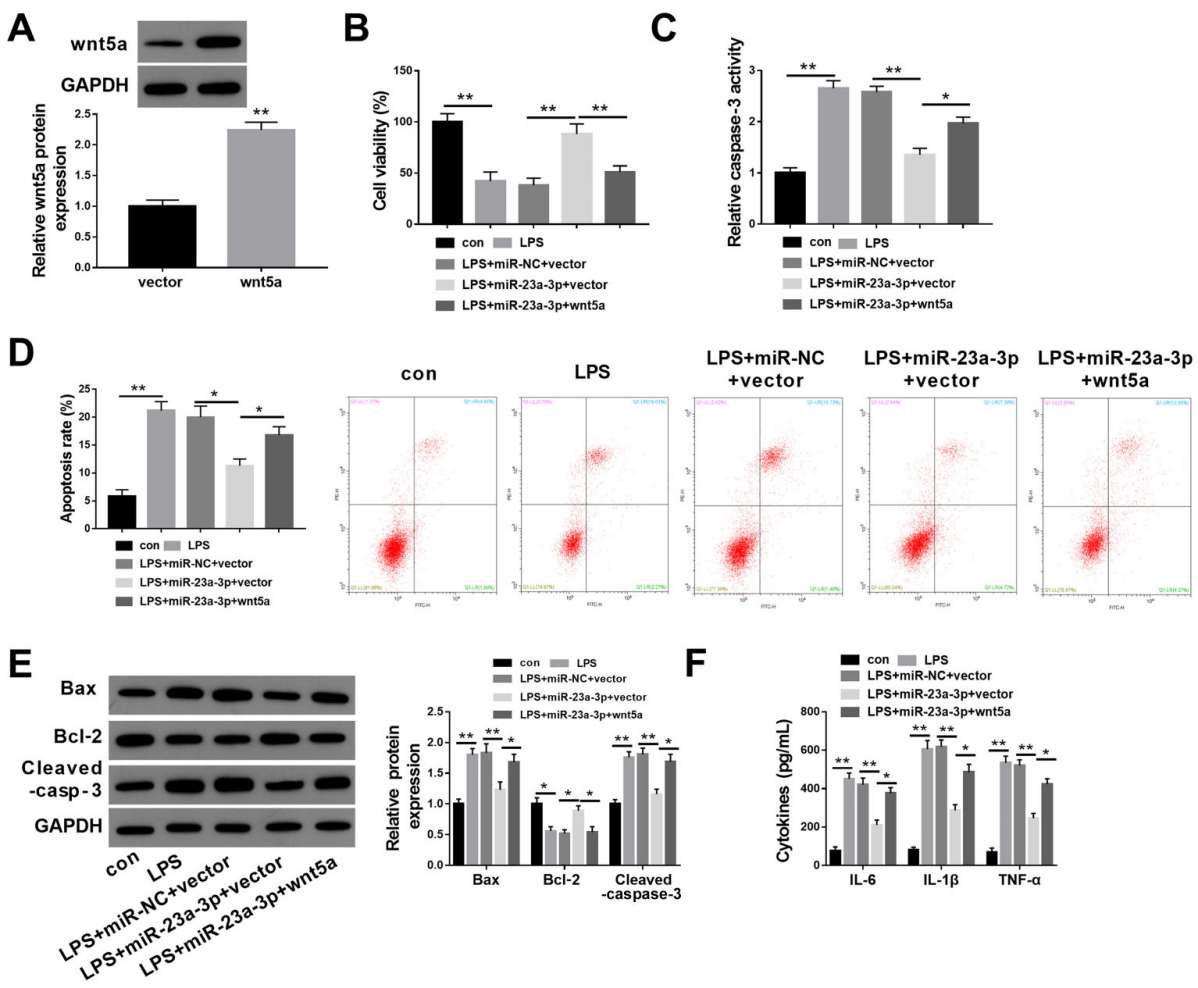
miR-23a-3p attenuates lipopolysaccharide (LPS)-induced HK-2 cell injury by downregulating wnt5a. **A**, Western blotting was conducted to detect the protein levels of wnt5a in HK-2 cells after the overexpression of wnt5a. Cell viability (**B**), caspase-3 activity (**C**), apoptosis rate (**D**), the expression of apoptosis-related proteins (**E**), and the production of cytokines (**F**) were determined in LPS-stimulated HK-2 cells after the overexpression of miR-23a-3p and wnt5a. The results are representative of three independent experiments (n=3 per group). Data are reported as means±SD. *P<0.05; **P<0.01 (ANOVA). con: control; NC: negative control.

To further investigate whether the influence of miR-23a-3p on LPS-induced cell injury was achieved by regulating the expression of wnt5a in HK-2 cells, the expression of wnt5a was successfully overexpressed by transfection with pcDNA-wnt5a as demonstrated by western blotting (Figure 4A). The co-overexpression of miR-23a-3p and wnt5a significantly reversed the effects of miR-23a-3p overexpression on cell viability (Figure 4B), apoptotic rate (Figure 4C), expression of apoptosis-related proteins (Figure 4D), caspase-3 activity (Figure 4E), and the production of cytokines (Figure 4F) in LPS-stimulated HK-2 cells. These results suggested that miR-23a-3p upregulation enhanced LPS-induced injury in HK-2 cells by the suppression of wnt5a.

### miR-23a-3p inhibited the activation of Wnt/&mac_bgr;-catenin signaling pathway in LPS-treated HK-2 cells

**Figure 5:**
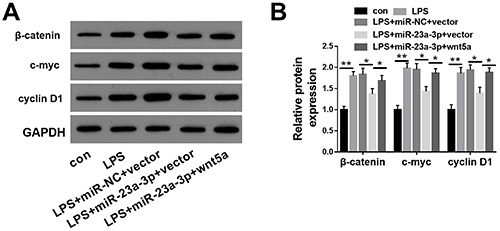
miR-23a-3p inhibits the activation of Wnt/β-catenin signaling pathway in lipopolysaccharide (LPS)-treated HK-2 cells. **A** and **B**, Western blotting was conducted to detect the protein levels of β-catenin, c-myc, and cyclin D1 in LPS-stimulated HK-2 cells after the overexpression of miR-23a-3p and wnt5a. The results are representative of three independent experiments (n=3 per group). Data are reported as means±SD. *P<0.05; **P<0.01 (ANOVA). con: control; NC: negative control.

On the basis of the critical role of the Wnt/β-catenin signaling pathway in kidney injury, we examined whether miR-23a-3p inactivated Wnt/β-catenin signaling pathway. The protein levels of Wnt/β-catenin signaling pathway-related proteins including β-catenin, c-myc, and cyclin D1 were then detected. Western blotting analysis consistently confirmed that the expression levels of β-catenin, c-myc, and cyclin D1 were elevated in LPS-treated HK-2 cells, whereas the restoration of miR-23a-3p expression inhibited the activation of Wnt/β-catenin signaling, which was significantly reversed after wnt5a overexpression (Figure 5A and B).

## Discussion

The results of the current study showed that miR-23a-3p expression was suppressed in patients diagnosed with sepsis-associated AKI and in LPS-induced HK-2 cells. In addition, overexpression of miR-23a-3p alleviated LPS‐associated injury in HK-2 cells. Our results further revealed the interaction between miR‐23a‐3p and wnt5a, and miR-23a-3p overexpression restrained LPS‐induced cell injury by suppression of wnt5a expression. Finally, we demonstrated that miR-23a-3p upregulation could mediate LPS‐associated cell injury by inactivating Wnt/β-catenin signaling pathway.

Recently, accumulating evidence has strongly implied that a few miRNAs have been characterized as diagnostic biomarkers in the pathogenesis of sepsis and related complications, including miR-21 (20), miR-214 (21), and miR-122 (22). Microarray assay has shown that miR-23a-3p was significantly downregulated in sepsis-induced AKI patients compared with the healthy controls. In previous reports, miR-23a-3p has been reported to be abnormally expressed in diverse tumor types and possesses oncogenic or tumor-suppressing functions. For example, Ma et al. (23) demonstrated that miR-23a-3p mitigated mucosal melanoma growth, migration, invasion, and tumorigenicity by targeting ADCY1 via the suppression of cAMP and MAPK signaling pathways. Chen et al. (24) showed that miR-23a-3p inhibited the proliferation and invasion of oral squamous cell carcinomas cells, while inducing apoptosis by decreasing FGF2 expression. Xiang et al. (25) reported that miR-23a-3p might target PCDH17 to accelerate the proliferation and G1/S cell cycle transition in hepatocellular carcinoma. The present study demonstrated for the first time that the expression of miR-23a-3p was significantly downregulated in serum of patients with sepsis-induced AKI and LPS-induced septic AKI cell model. These data suggested that the aberrant expression of miR-23a-3p might be associated with the progression of septic AKI.

The pathogenesis of septic AKI is associated with the apoptosis of kidney tubular epithelial cells and inflammatory injury (26-28). Consistently, this research implied that LPS treatment inhibited cell viability and increased apoptosis and the levels of inflammatory cytokines IL-6, IL-1β, and TNF-α in HK-2 cells, while miR-23a-3p upregulation repressed these biological behaviors of HK-2 cells under LPS-stimulated conditions. These results showed that miR-23a-3p might be implicated in the occurrence and development of septic AKI by regulating cell apoptosis and inflammation.

Further investigations were performed to elucidate the downstream mechanism of miR-23a-3p in sepsis-induced AKI. Bioinformatics and dual luciferase analysis confirmed that wnt5a, a noncanonical Wnt ligand, was a direct target of miR-23a-3p, the expression of which was negatively correlated with miR-23a-3p expression in patients with septic AKI. It has been demonstrated that Wnt5a plays an emerging role in hyperuricemia-induced kidney tubular injury (29). Also, Wnt5a possesses pro-inflammatory properties in the pathogenesis of inflammatory diseases (30), including sepsis (31,32). Inspired by the miRNA regulatory network, we observed that the overexpression of wnt5a could reverse the effects of miR-23a-3p in HK-2 cells treated with LPS. Further studies demonstrated that targeting of wnt5a by miR-23a-3p resulted in the inhibition of Wnt/β-catenin axis in LPS-treated HK-2 cells.

We concluded that miR-23a-3p suppressed the development of sepsis-induced AKI by downregulating Wnt/β-catenin pathway, at least in part, via targeting wnt5a. Our findings might provide a novel therapeutic strategy for sepsis-associated complications.
